# Myofibroblast modulation of cardiac myocyte structure and function

**DOI:** 10.1038/s41598-019-45078-2

**Published:** 2019-06-20

**Authors:** Chandan K. Nagaraju, Eef Dries, Guillaume Gilbert, Mouna Abdesselem, Nan Wang, Matthew Amoni, Ronald B. Driesen, Karin R. Sipido

**Affiliations:** Department of Cardiovascular Diseases, Division of Experimental Cardiology, KU Leuven, Campus Gasthuisberg O/N1 Box 704, Herestraat 49, Leuven, B-3000 Belgium

**Keywords:** Electrophysiology, Cardiovascular diseases

## Abstract

After myocardial infarction, resident fibroblasts (Fb) differentiate towards myofibroblasts (MyoFb), generating the scar tissue and the interstitial fibrosis seen in the adjacent myocardium. Fb and MyoFb have the potential to interact with cardiac myocytes (CMs) but insight into the phenotype-specific role and mode of interaction is still incomplete. Our objectives are to further define the modulation of CMs by MyoFbs compared to Fbs, as well as the role of direct contact through gap junctions vs. soluble mediators, using Fbs and CMs from pig left ventricle. Fbs were treated to maintain an undifferentiated state (SD-208) or to attain full differentiation to MyoFb (TGF-β1). Fbs and MyoFbs were co-cultured with CMs, with the possibility of direct contact or separated by a Thincert membrane. Only in direct co-culture, both Fbs and MyoFbs were able to decrease CM viability after 2 days. Only MyoFbs induced significant distal spreading of CMs in both direct and indirect co-culture. MyoFbs, but not Fbs, readily made connections with CMs in direct co-culture and connexin 43 expression in MyoFb was higher than in Fb. When coupled to CMs, MyoFbs reduced the CM action potential duration and hyperpolarized the CM resting membrane potential. Uncoupling reversed these effects. In conclusion, MyoFbs, but not Fbs, alter the CM structural phenotype. MyoFbs, but not Fbs, are likely to electrically connect to CMs and thereby modulate the CM membrane potential. These data provide further support for an active role of MyoFbs in the arrhythmogenic substrate after cardiac remodelling.

## Introduction

Cardiomyocytes (CMs) make up the bulk of the volume and mass of the heart, and provide its pumping capacity. Yet the heart also contains numerous non-CM cells such as vascular cells and fibroblasts (Fbs). Fbs serve a structural function in the heart, by providing the extracellular matrix structure and interacting with CM through direct connections and via paracrine signaling^1^. Furthermore, Fbs are important during the cardiac remodeling process under increased load, through mechanosensing, secreting proteins e.g. angiotensin and endothelin^2–4^, and communicating with cardiac myocytes through exosomes^5,6^. The differentiation of Fbs into myofibroblasts (MyoFbs) is at the basis of fibrosis and matrix remodeling during disease. Scar formation is part of the healing process after myocardial infarction (MI), but interstitial fibrosis, particularly in the border zone, contributes to the substrate for arrhythmias by slowing conduction and creating re-entry circuits^7^.

In co-cultures *in vitro*, Fbs and CMs can make direct contacts, with Fbs electrotonically modulating the membrane potential of CMs. Several *in vitro* studies have examined this process in more detail, with some of these *in vitro* models mimicking the Fb-CM interactions in pathology such as atrial fibrillation or MI^8–12^. Limitations of these studies include the difficulties of culturing CMs and Fbs, as culture induces phenotypic alterations. Often, neonatal myocytes are used, since they are amenable to growth and proliferation while maintaining a cardiac phenotype, although not that of an adult myocyte. Fbs in culture are well known to differentiate and most often therefore the model is one of (mixed) MyoFb phenotypes^13^.

In the heart *in situ*, direct coupling through gap junctions between Fbs and CMs were first described for the sino-atrial node^14^. A recent report described close interactions of Fb extensions and collagen at T-tubules of ventricular myocytes level but did not include data on gap junctions^15^. Such connections, even if rare, can be seen in cardiac pathology^16–18^. It is thus likely that the interactions *in vivo* will be mostly in pathology when Fbs are differentiated into MyoFbs. Recently, Cartledge *et al*., compared the influence of freshly isolated Fbs and cultured Fbs, as well as Fbs isolated from pressure-overloaded rat hearts, on adult CMs, bringing the conditions closer to the *in vivo* situation^12^. In these experiments, direct contact was inhibited and the effects on myocyte viability and function resulted from soluble mediators^12^. The authors could show that differentiated MyoFbs have more pronounced effects, reducing viability and modulating calcium handling of the myocytes.

In the present study, we take advantage of our previous work where we demonstrated that we could grow rat cardiac Fbs with different phenotypes^19^. Our objectives are to define the role of the Fb phenotype as well as the role of direct contact through gap junctions compared to soluble mediators for cells derived from the pig left ventricle (LV). Interest in this animal model stems from its use to study remodelling after myocardial infarction (MI) and the impact on cardiac function and arrhythmias. We have previously reported that after MI fibroblastic cells in the area near the MI scar are well-differentiated MyoFbs^20^. Therefore, here we compare the impact on CMs of co-culturing with MyoFbs compared to Fbs, and we compare modulation through direct contact, to indirect contact with cells separated by a Thincert. A schematic of the experimental design is shown in Supplemental Figure S1.

## Results

### Phenotype of fibroblasts and myofibroblasts derived from pig left ventricle

**Figure 1:**
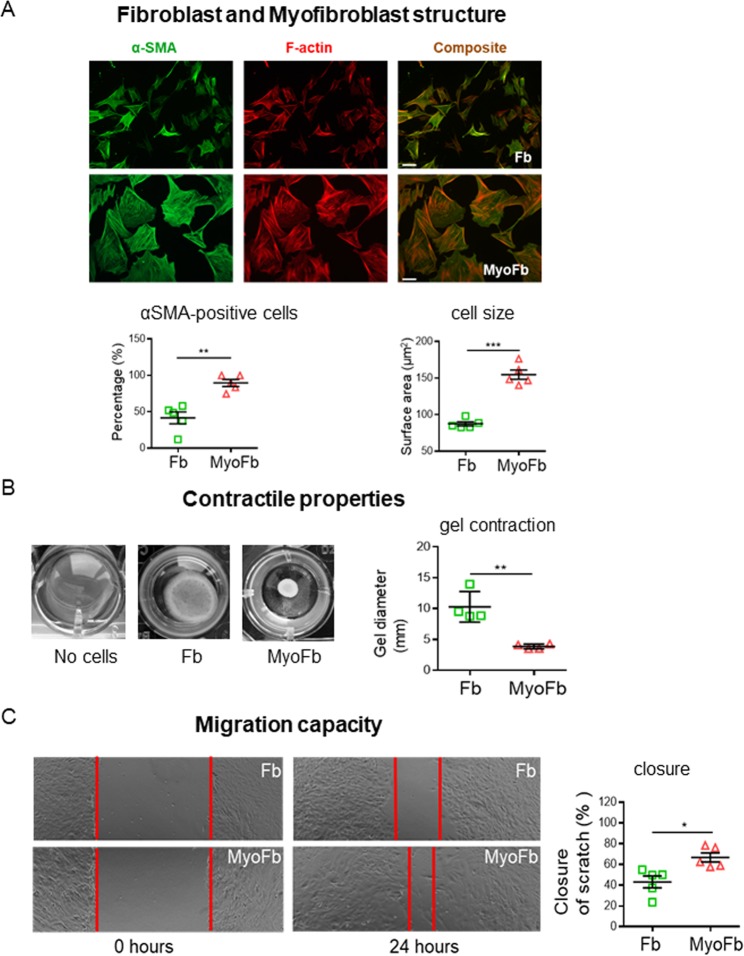
Fb phenotypes characterization. (**A**) Example of cultures of Fbs and MyoFbs with cells stained for F-actin and α-SMA. Quantification of cells positive for α-SMA, as percentage of all cells, and data of average cell size, measured in F-actin staining (N cultures = 5). (two-tailed Student t-test, **p = 0.00978, ***p = 0.00012). (**B**) Analysis of the collagen gel contraction after 48 hours in culture. (N cultures = 4; two-tailed Student t-test, **p = 0.00218). (**C**) Representative image of scratch at 0 hours and after 24 hours and analysis of cell migration of Fbs after 24 hours. (N cultures = 4; two-tailed Student t-test, *p = 0.01103).

Compared to Fbs (treated with SD-208 to prevent differentiation), MyoFbs (treated with TGF-β1 to promote differentiation) are larger and have a prominent network of actin stress fibres decorated with α-smooth muscle actin (Fig. 1A). This latter difference results in a larger contractile capacity of MyoFbs (Fig. 1B) and migration capacity (Fig. 1C).

One of the prominent features of MyoFbs in the diseased myocardium is to alter collagen synthesis and participate in reparative and reactive fibrosis as illustrated in Supplemental Figure S2. *In vitro*, MyoFbs have the highest capacity to synthesize (0.8 ± 0.05 µg/1000 cells, pellet) and secrete collagen (0.09 ± 0.04 µg/mL, in the medium), when compared to Fbs, as can also be appreciated from Sirius red staining of the cultures. Immunostaining for lysyl oxidase (Lox), which is a key regulator of collagen cross-linking^21^, is also higher in MyoFbs compared to Fbs.

### Cardiomyocyte adaptation induced by different fibroblastic phenotypes

**Figure 2:**
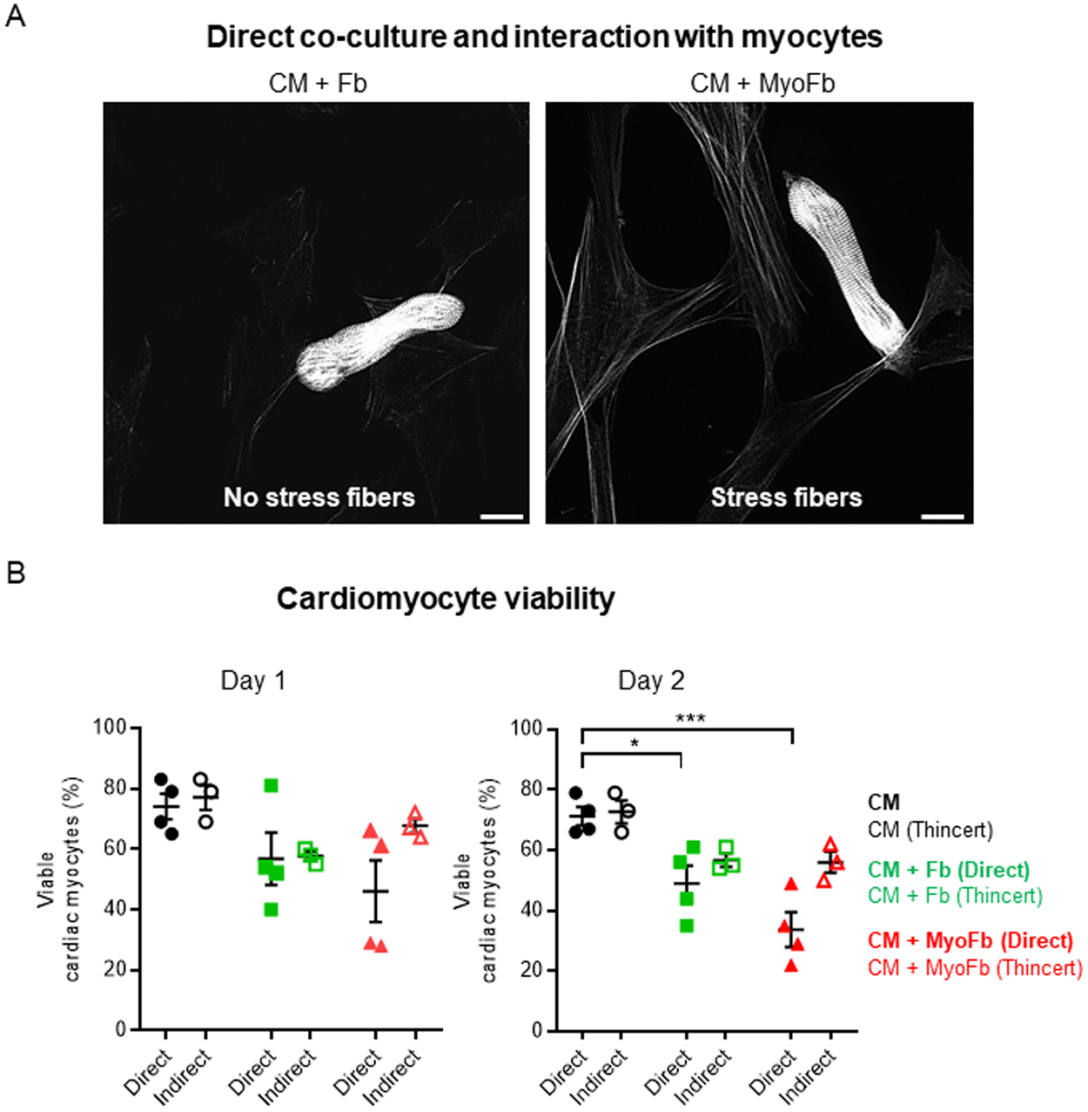
Co-culture of CMs with Fbs and MyoFbs reduces CM viability. (**A**) Example of direct co-cultures of CMs with Fbs (left) and MyoFbs (right) after fixation and staining for F-actin. Stress fibers are visible in the MyoFbs but absent in the Fbs. (**B**) Percentage of live CMs after 24 and 48 hours in direct and indirect (Thincert) co-culture. (N cultures = 4 for direct and 3 for indirect; two-way ANOVA with Bonferroni post hoc test *p = 0.0344, ***p = 0.0003).

**Figure 3:**
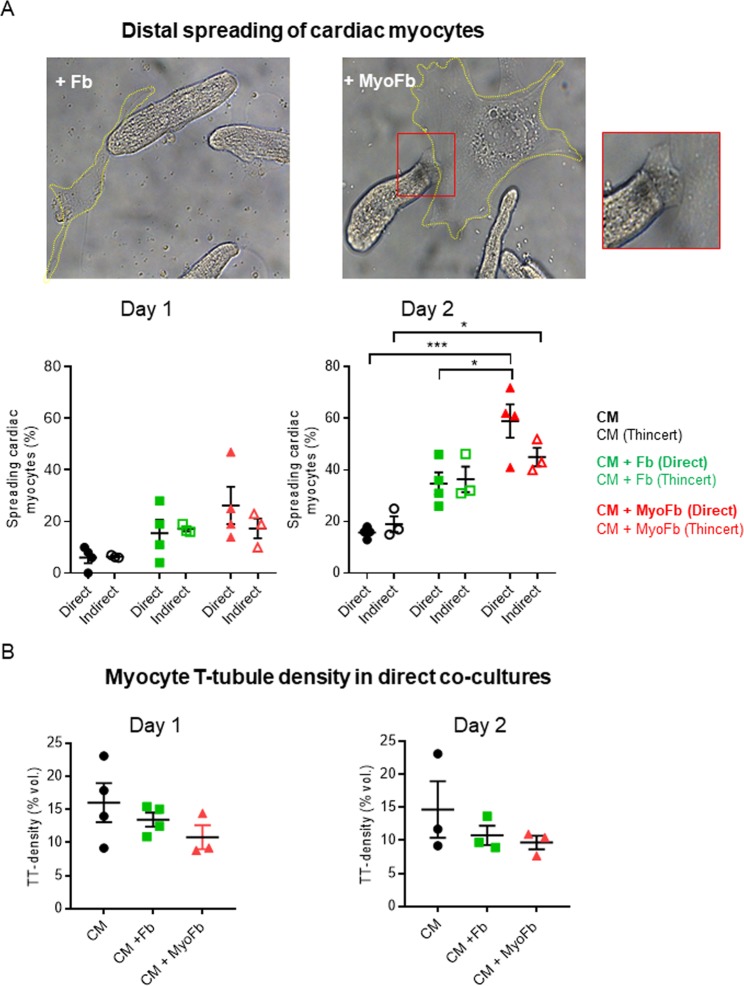
Distal spreading of CMs in the presence of MyoFbs. (**A**) Light microscopy images of co-cultures and detail of distal spreading in the presence of MyoFbs. Percentage of CMs with distal spreading after 24 and 48 hours in direct co-culture and with Thincert/indirect co-culture. (**B**) Quantification of potential changes in CM T-tubule density in direct co-culture. (n cells = 15–20; N culture 3–4; two-way ANOVA with Bonferroni post hoc test *p = 0.0227 for top, *p = 0.0123 for bottom, ***p = 0.000032).

In cultures without Fbs or MyoFbs, around 75% of plated CMs remained viable at 1 and 2 days of culture (Fig. 2A,B). In Fb or MyoFb direct co-cultures, CM cell death was significantly increased at day 2, which was not seen in indirect co-cultures (Fig. 2B). When cultured alone, the majority of CMs maintained their rod-shaped phenotype within the first 24 hours, though distal spreading was seen in 20% of cells after 2 days (Fig. 3A). In co-cultures, spreading of CMs was most pronounced after 2 days in culture and was significant only for MyoFbs (Fig. 3A). We analyzed T-tubule (TT) density of the rod-shaped CMs. In simple CM cultures, TT density remained similar to freshly isolated cells even after day 1 and day 2 (Supplemental Figure S3A). Neither Fbs nor MyoFbs, in direct co-culture, induced a significant change in TT density (Fig. 3B). A representative example for membrane stained CM in co-culture with MyoFb is shown in Supplemental Figure S3B.

### Myofibroblasts in co-culture with cardiomyocytes alter the myocyte action potential duration

**Figure 4:**
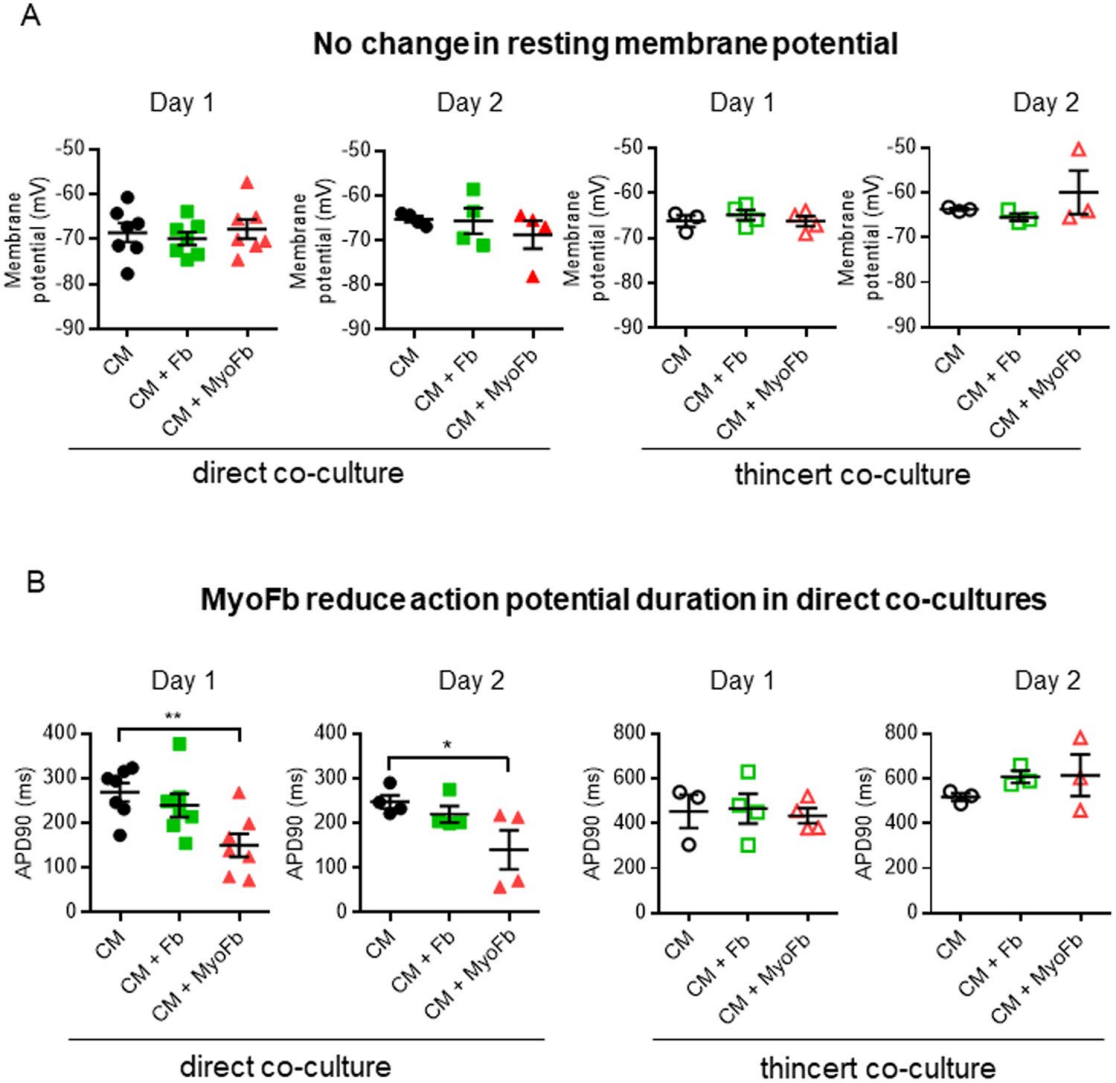
Modulation of the membrane potential of CMs by MyoFbs. (**A**) Resting membrane potential. (**B**) Action potential duration at 90% repolarization (APD_90_). CMs in direct and indirect (thincert) co-culture with Fbs or MyoFbs. (n cells = 10–35; N culture = 3–7; 1-way ANOVA with Bonferroni post hoc test, **p = 0.006, * = 0.0137).

The possibility that MyoFbs modulate the membrane potential of CMs, directly or through soluble mediators, was investigated by studying the action potential (AP) profile of CMs in the presence of different Fb phenotypes and culturing conditions. No differences were observed in the resting membrane potential (RMP) of CMs in the different groups at day 1 and day 2 of direct or indirect co-cultures (Fig. 4A). However, the AP duration (APD), measured as the duration to 90% repolarization, APD_90_, of CMs co-cultured with MyoFbs at 1 day and day 2 was on average lower than in the CMs (Fig. 4B). This was not the case in indirect co-culture (Fig. 4B).

### MyoFbs more readily make functional couplings with cardiomyocytes

**Figure 5:**
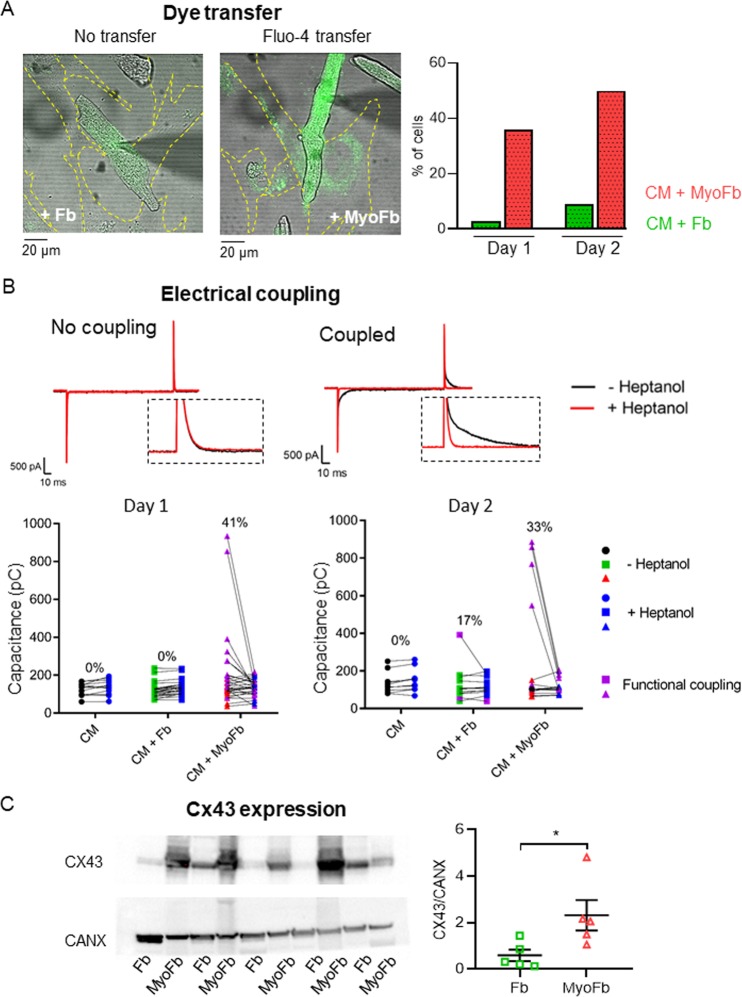
Identification of functional coupling of CM with (Myo)Fbs in direct co-culture. (**A**) Example of studying dye transfer (Fluo-4) between CM and Fb (no transfer) or MyoFb (transfer) and mean data at day 1 and day 2 in direct co-culture (n cells = 11–31; N culture = 3–5). (**B**) Examples and mean data of cell membrane capacitance measurement of CMs before and after heptanol application. Changes in cell capacitance larger than 10 pF are shown in purple and indicate functional couplings between CMs and Fb phenotype (n cells = 10–30; N culture = 3–5). (**C**) Connexin 43 protein blot and expression in cell lysates. (N = 5; two-tailed Student t-test, *p = 0.039).

We further assessed the presence of functional gap junction coupling with different Fb phenotypes in direct co-cultures. We first studied permeation by measuring the transfer of Fluo-4 dye (Fig. 5A). Voltage-clamped CMs in whole-cell configuration were loaded with Fluo-4 through the patch pipette. The transfer of Fluo-4 from CMs to Fbs was quantified after 5 minutes at resting membrane potential (Fig. 5A). In the Fb co-cultures, we saw Fluo-4 transfer to neighbouring Fbs in less than 10% of CMs. In MyoFbs co-cultures, such transfer was seen in 50% of CMs.

Electrically coupled cells will behave as a single electrical capacitance. Therefore, we measured cell capacitance transients using a 10 mV hyperpolarizing pulse before and after heptanol application (Fig. 5B). By uncoupling the Fb/MyoFb-CM interaction with heptanol, we expect the capacitance to decrease. This is indeed what occurred in 41% and 33% of CMs co-cultured with MyoFbs at day 1 and day 2, respectively. No functional couplings were found between CMs and Fbs at day 1 and only 17% of the cells showed a functional coupling at day 2.

MyoFbs have an increased expression of connexin 43 (Cx43), facilitating the formation of gap junctional coupling with CMs (Fig. 5C).

### MyoFbs functionally coupled to cardiomyocyte alter their resting membrane potential and action potential duration

**Figure 6:**
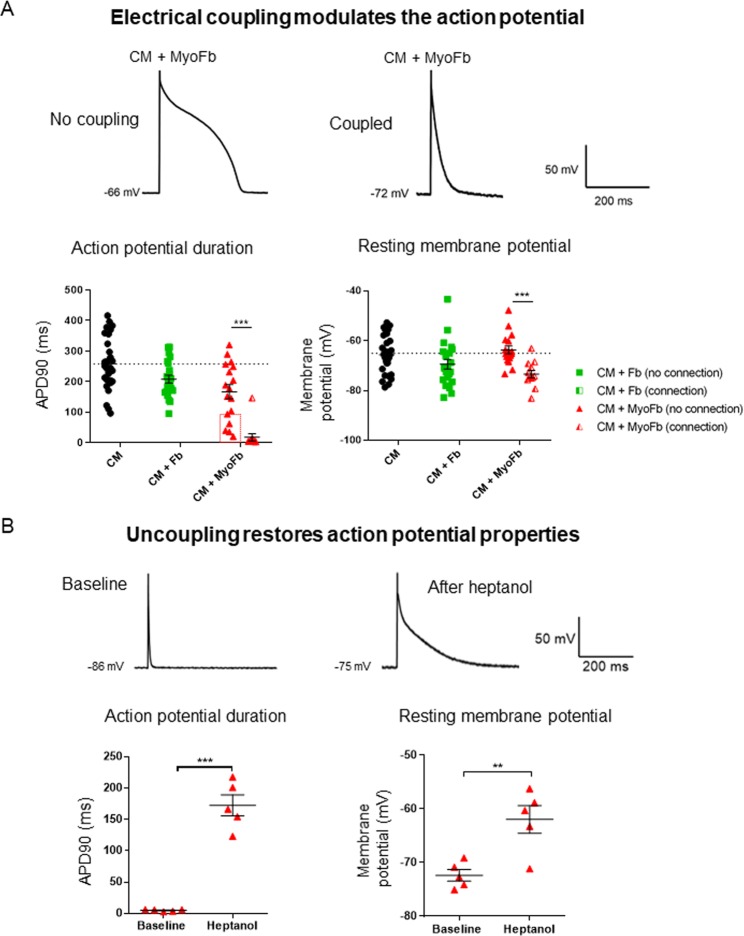
Electrical coupling to MyoFbs hyperpolarizes CMs and reduces action potential duration. (**A**) Examples of APs in CMs in direct co-culture with MyoFbs, with and without electrical coupling as determined by the capacitance test of Fig. 5. Mean data of ADP_90_ and RMP for different co-culture conditions at day 1 (n cells = 12–30; N culture = 5; 2-way ANOVA with Bonferroni post hoc test, ***p = 0.0002). (**B**) Examples of AP in CMs before and after heptanol and with functional coupling to MyoFb. Mean data of APD_90_ and RMP of CMs before and after heptanol and with functional coupling to MyoFb (n cells = 5, a subset of data in panel A; paired two-tailed Student t-test, **p = 0.008, ***p = 0.0006).

The presence of functional electrical coupling predicts that MyoFbs will affect the membrane potential of CMs. We studied this prediction comparing RMP and APD_90_ for CMs with and without coupling, as determined by dye transfer and by the reduction of the cell capacitance after heptanol application. When CMs are functionally coupled to MyoFbs, the RMP is hyperpolarized and APD_90_ is significantly shorter than in cells without coupling (Fig. 6A). In 5 out of 17 cells dye transfer or capacitance measurements could not provide evidence for electrical coupling to MyoFbs (lower values in Fig. 6A marked by a square) yet the APD_90_ was below 100 ms.

After heptanol, some cells developed leak currents and were lost for follow-up of AP properties. From the data set of Fig. 6A, AP data after heptanol were obtained in 5 CMs with an established coupling. In these, heptanol restored the RMP and APD_90_ to values comparable to that of baseline values in non-coupled CMs (Fig. 6B). Data were also obtained in 3 CMs that were classified as not connected yet had an APD shorted than 100 ms. In these CMs application of heptanol also induced a prolongation of the APD, suggesting they were also coupled to MyoFbs. In 4 cells that were classified as not connected and had a baseline APD between 150 and 290 ms, there was no prolongation and in contrast APD was reduced in these cells, in line with previously reported effects of heptanol on AP properties^22^ and the reduction of Ca current by heptanol^23^.

We extended the data set using Fbs that were cultured in the absence of SD-208 and spontaneously differentiated into MyoFbs, as shown by the presence α-SMA and contractile capacity (Supplemental Figure S4A). These spontaneously differentiated MyoFbs also decreased CM viability and induced spreading (Supplemental Figure S4B). Functional couplings were present in 30% of CMs and these CMs had a short APD (Supplemental Figure S4C).

**Figure 7:**
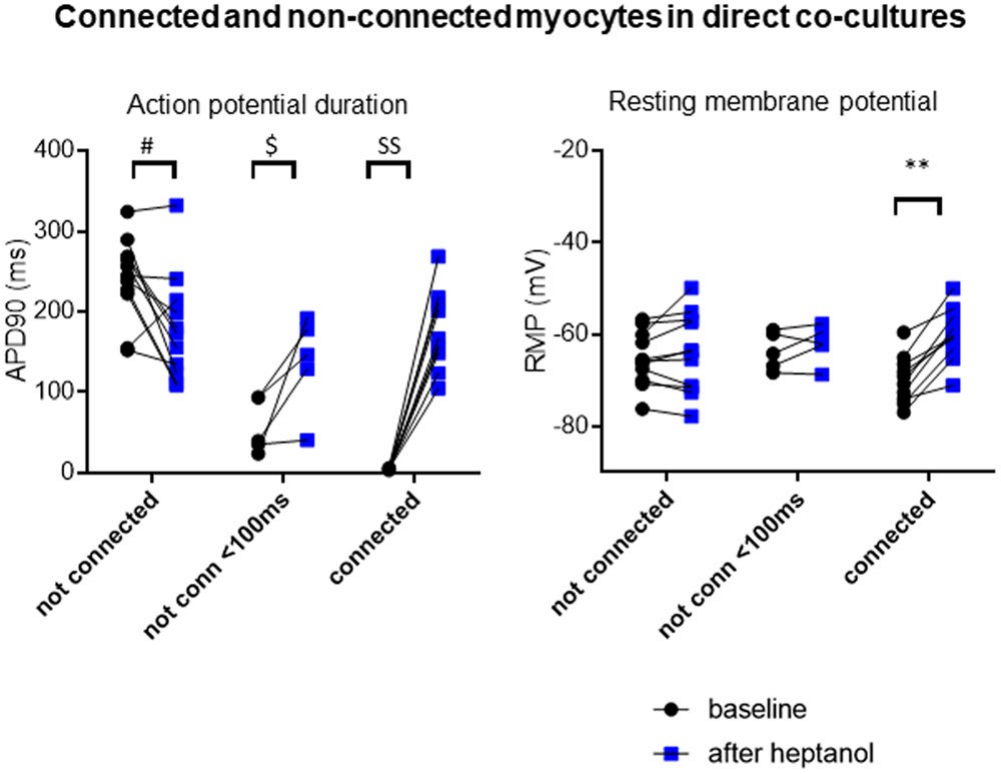
Heptanol prolongs action potential in CMs coupled to MyoFbs. Pooled data from 8 co-cultures of CMs with MyoFbs (spontaneously differentiated or TFGβ1-treated), showing paired data before and after heptanol treatment. CMs were categorized based on the change in capacitance after heptanol as not connected (n = 11), not connected but with short APD (n = 5), or connected (n = 10). In the first group, APD overall shortened (^#^p = 0,01318), in the second group APD prolonged (^$^p = 0.03980) and in the third group as well (^$$^p < 0.00001). For the RMP, only the third groups had a statistically significant depolarization (**p = 0.00005).

Figure 7 provides pooled data on all CM AP properties before and after heptanol for direct co-cultures with MyoFbs: CMs where the capacitance did not increase after heptanol and with an APD >100 ms, CMs where the capacitance did not increase after heptanol and with very short AP, and CMs with a clear increase of capacitance after heptanol. The increase in APD after heptanol is statistically significant in the CMs in the last 2 groups, suggesting all of these CMs are coupled to MyoFbs. Changes in the resting membrane potential are small and depolarization of the resting membrane potential was significant only in the third group.

## Discussion

The main findings of the present study are that the modulation of CMs by activated MyoFbs is distinct from that by non-activated Fbs. In contrast to Fbs, activated MyoFbs are likely to make direct functional contacts with CMs and thereby hyperpolarize the RMP and shorten the AP.

### MyoFbs have a higher potential to functionally couple to cardiomyocytes

Activated MyoFbs have a high potential to form functional couplings with CMs compared to normal Fbs. This is in line with the work by Chilton *et al*., where functional couplings were found in 58% of the adult rabbit CMs when cultured with *in vitro* differentiated MyoFbs^10^. This increased fraction of couplings in the MyoFb phenotype may reflect the stronger migration potential of MyoFbs as well as the higher expression of Cx43 compared to Fbs. Yet, the vicinity of Fbs to CMs is not different as seen in the example of Figs 2A and 3A, indicating that the potential for creating functional gap junctions is a distinctive property of MyoFbs. These data are in line with recent studies reporting functional junctions between CM and non-myocyte in infarct and peri-infarct regions after MI^16,18^.

### MyoFbs functionally coupled to CMs affect electrical excitability

On an aggregate level, without taking into account the presence of functional couplings, there was no difference in myocyte RMP when co-cultured with different Fb phenotypes (Fig. 4). This is in line with the study from Chilton *et al*.^10^. However, when subcategorizing CM profiles based on the presence of a functional coupling with Fbs, our data showed that functionally coupled activated MyoFbs induced a significant increase in the CM RMP (i.e. hyperpolarization) compared to control CMs (Fig. 6). This was somewhat surprising as previous modeling studies predicted a depolarization of the CM membrane when connected to Fbs^24^. A potential explanation for this could be the higher polarization of the MyoFbs in the present study compared to the Fbs properties used in the computational models^24,25^. Previous studies reported the RMP in neonatal and atrial cardiac Fbs to be less negative (between –40 mV and −20 mV) compared to myocytes (between −65 mV and −80 mV)^26,27^. Consequently, the functional coupling between these cell types would result in a modest depolarization of the CM membrane. Other studies have reported the presence of inwardly rectifying potassium currents (K_ir_) in Fbs, which act as a primary determinant of the RMP, and with higher levels of I_K1_, the Fb RMP was more (hyper)polarized, between −80 and −70 mV^28^. The presence of K_ir_ channels in activated MyoFbs and not in Fb, could explain the hyperpolarization in CMs upon functional coupling with these cells in the present study. This is in line with previous work showing increased expression of KCNJ2 with larger inward-rectifier current in atrial Fbs/MyoFbs during cardiac remodelling^29,30^.

In addition to the hyperpolarized RMP, functionally coupled CMs had a triangular AP with short plateau phase and fast repolarization (Fig. 6). These features are indicative for a passive loading and modulation by a non-excitable, polarized cell. Indeed, when functional couplings between CMs and Fbs are disrupted upon heptanol application, CM excitability is restored, with less hyperpolarized RMPs. Therefore, the hyperpolarization and APD_90_ shortening in CMs is likely based on the junctional current flow from hyperpolarized activated MyoFbs, rather than by the influence of paracrine factors. CMs cultured with MyoFbs separated by a Thincert, had no change of RMP or APD, supporting the direct junctional current flow hypothesis. A number of CMs in direct co-culture without apparent functional coupling but short APD also had longer APD after heptanol suggesting they had MyoFb-CM connections, not detected with the capacitance test. A paracrine effect from MyoFbs at close distance in direct co-cultures is not likely to be involved as this would lead to generalized APD shortening of all CMs given the density of MyoFbs in the co-cultures.

### Mechanistic implications for arrhythmogenesis

It was previously thought that cardiac fibrosis, i.e. the collagen accumulation, was the main cause for reentrant arrhythmias due to the lack of electrical impulse propagation. However, this paradigm has evolved^31^. (Myo)Fbs and CMs both express connexons allowing formation of gap junctions between the two cell types^16^ and the present data bring additional support to a role for these cells through direct coupling in a different species, and demonstrating hyperpolarization of CMs by MyoFbs.

Electrical heterocellular MyoFb-CM couplings may allow delayed conduction, rather than no conduction, as demonstrated also by others in co-cultures^32^. The impact of MyoFb is large because of the relatively large size and capacitance of the MyoFbs compared to Fbs, allowing considerable electrotonic influence on CMs. This interaction will be of particular importance in the MI border zone, where direct contacts between MyoFbs and CMs have been documented^33^, with recent elegant experiments recording conducted APs in MyoFbs^18^. Direct contacts can be established in CMs after loss of the glycocalyx and could result from increased MMP production in the border zone^34^. Slowed and discontinuous electrical propagation within the MI border zone can thus form a potential substrate for micro-reentry and ventricular arrhythmias. The shortening of the APD can further enhance APD dispersion within the myocardium during post-MI remodeling with reduced repolarization reserve. Lastly, in contrast to previous data from neonatal Fb-CM couplings, we did not observe triggered activities or automaticity in our co-culture conditions^32^. In these studies, neonatal Fb-CM couplings induced a more depolarized RMP in CMs, thereby promoting an increased probability of after depolarizations and triggered APs. These observations are less likely to occur in the present co-culture of adult pig cells where MyoFb coupling induced hyperpolarization in CMs.

### Limitations

The classification of the functional Fb-CM couplings in this study was based on the experiments with the gap junction uncoupler heptanol. For this, functional Fb-CM couplings were considered when at least a 10 pF decrease in capacitance was induced upon heptanol application. This might only reveal the presence of large functional Fb-CM couplings and would not detect smaller Fb-CM connections. Furthermore, high resolution and/or electron microscopy studies would be needed to further unravel the structural aspects and connection targets of these functional Fb-CM connections. A recent study by Crossman *et al*., showed the presence of Fb filopodia within the lumen of enlarged T-tubules in heart failure tissue^15^.

Changes in the CM electrophysiological profile were significant after 24 hours of co-culture. Longer co-culture periods (48 h) showed similar trends, but the number of observations was smaller and some trends did not reach statistical significance. This limitation is related to the higher rate of cell death after 48 hours of co-cultures, hampering collection of large data sets.

In the present study we did not study the impact of MyoFb-CM connections on contractile properties due to technical limitations in our experimental setup.

## Conclusions

In cardiac remodeling, as in the myocardium adjacent to MI, the presence of activated MyoFbs will affect myocyte structure and function, and thereby facilitate arrhythmias. Non-differentiated Fbs do not modulate myocyte function. As MyoFbs retain plasticity and can revert to undifferentiated state^19^ this opens perspectives for therapeutic interventions.

## Methods

### Animal care

Healthy pigs of both sexes (40–45 kg) were housed and treated according to the Guide for the Care and Use of Laboratory Animals (National Institute of Health, U.S.A.). Experimental protocols were approved by the in-house ethical committee P110/2016 (Ethical Committee for Animal Experiments, KU Leuven). Animals were pre-anesthetized with tiletamine/zolazepam (8 mg/kg), sacrificed with an overdose of pentobarbital (100 mg/kg) under artificial ventilation followed by quick removal of the heart.

### Cell isolation, preparation and characterization of Fbs and MyoFbs

Fbs from the LV were isolated by a chunk enzymatic digestion protocol as described before^20^. After first passage, Fbs were seeded at a density of 3000 cells/cm^2^ and cultured in DMEM (ThermoFisher) medium with 10% foetal bovine serum (FBS) and 1% of penicillin/streptomycin (Invitrogen). To induce specific Fb phenotypes, cells were either treated for 6 days with recombinant human TGF-β1 (400 pmol/L; PeproTech) or with SD-208 (3 μmol/L; Sigma-Aldrich), a specific TGF-β-receptor-I (TGF-β-RI) kinase inhibitor to respectively promote and suppress differentiation. After 6 days, the phenotype was assessed by protein expression and morphology, and functionally as the capacity for proliferation, migration, and contraction of a floating gel. These pre-treated Fbs and MyoFbs were then processed for co-culture with freshly isolated CMs. The supplemental material and Figures S1 detail the preparation and properties of the fibroblastic cells and the subsequent setup of the co-cultures.

### Co-culture set up

CMs were isolated enzymatically from LV as described before^35^. After isolation, CMs were pre-plated on a T-75 culture flask in Medium 199 (ThermoFisher Scientific) with 1% of penicillin/streptomycin (ThermoFisher Scientific) and incubated for 4 hours so that the non-CM cell population could attach to the culture flask. The supernatant with the CMs was collected from the culture flask after 4 hours and CMs were plated onto laminin (10 µg/mL; Sigma) coated coverslips, in 6-well plates for 3 hours. For direct 2-D co-cultures, Fbs or MyoFbs were added to the 6-well plate, in a ratio of 2.5 (Fb/MyoFb):1 (CM). The previously obtained Fbs/MyoFbs were cultured in CM medium with 2.5% fetal bovine serum for 24 hours before they were seeded onto the CM culture. To set up 2-D indirect co-culture systems, we used a 0.4 µm Thincert™ (Greiner Bio-One) preventing direct contact of Fbs with CMs but allowing the Fb secretome to diffuse to CMs. After 24 and 48 hours of co-culture, we quantified viability of CMs using light microscopy (Olympus IX2-SLP with MotiCam 3 camera), by counting the density of rod-shaped striated CMs and expressing the count as percentage of the density at day 0. We further evaluated structural remodeling in the live cells by counting the fraction of CMs with lateral or distal spreading.

### Electrophysiological characterization

We measured AP properties of CMs in different co-cultures. Cells were constantly perfused with normal Tyrode’ solution at 37 °C (in mmol/L: NaCl 137, KCl 5.4, MgCl_2_ 0.5, CaCl_2_ 1.8, Na-HEPES 11.8, and glucose 10; pH 7.4) and patch pipettes (2–3 MΩ) (GB 200-8 P, Science Products) were filled with (in mmol/L): K-aspartate 120, NaCl 10, KCl 20, K-HEPES 10, MgATP 5, and K_5_Fluo-4 0.05; pH 7.2. Cell capacitance transients were recorded in the voltage-clamp mode (Axon 200B amplifier, Axon Instruments) during a 10 mV hyperpolarizing pulse (−70 mV to −80 mV for 150 ms) before and after heptanol (2 mM) application; cell capacitance was quantified by integrating the area under the curve of the transients. APs were recorded in the current-clamp mode during steady-state stimulation at 1 Hz (2 ms current injections). AP duration (APD) was quantified at 90% repolarization (APD_90_). Transfer of Fluo-4 from the CM to Fb/MyoFb was assessed in confocal imaging using a Zeiss LSM 510 confocal system. Transfer of Fluo-4 was assessed using a 40x/1.3 oil immersion objective during 488 nm excitation using an Argon laser. Fluo-4 images were combined with light microscopic images to contour cell edges.

### Transverse and axial tubules (TATS) density analysis

We incubated co-cultured cells in 20 µM di-8-ANEPPS (Thermo Fisher) and imaged the stained sarcolemma with a multi-beam array confocal microscope (Visitech Infinity-3). We collected z-stack images every 0.5 µm with a pixel size of 0.11 µm. No deconvolution was applied. We developed an ImageJ (NIH) routine in order to segment and quantify CM size and subcellular tubules density, expressed as a fraction of volume. Di-8-ANEPPS stains non-specifically all membranes. Since Fbs are very thin cells compared to CMs, we could select CMs within the layered cultures, focusing on the top 5 µm of the thicker CMs for imaging of TATS.

### Statistics

Student-t-test, one-way ANOVA or two-way ANOVA with Bonferroni correction was used for statistical analysis. All data are expressed as mean ± SEM. The investigator was blinded during image analysis.

## Supplementary information

Electronic supplementary material
