# Supplementary material for: Myofibroblast modulation of cardiac myocyte structure and function
Source: Sci Rep. 2019 Jun 20;9:8879. doi: 10.1038/s41598-019-45078-2 (PMC6586929; doi:10.1038/s41598-019-45078-2)
Supplement: Supplementary file 1 — Electronic supplementary material [file 41598_2019_45078_MOESM1_ESM.docx]

Electronic supplementary material

Myofibroblast modulation of cardiac myocyte structure and function

Chandan K. Nagaraju*, Eef Dries*, Guillaume Gilbert, Mouna Abdesselem, Nan Wang, Matthew Amoni, Ronald B. Driesen and Karin R. Sipido#

Department of Cardiovascular Diseases, Division of Experimental Cardiology, University of Leuven, KU Leuven, Campus Gasthuisberg O/N1 Box 704, Herestraat 49, Leuven B-3000, Belgium

* Equal contribution

Karin R. Sipido, MD, PhD

Department of Cardiovascular Sciences, Division of Experimental Cardiology

KU Leuven

Campus Gasthuisberg, Herestraat 49

B-3000 Leuven, Belgium

E-mail: [karin.sipido@kuleuven.be](mailto:karin.sipido@kuleuven.be)

Supplemental methods

**Immunofluorescence characterization of Fb/MyoFb**

Immunostaining was performed on cultured cells to identify alpha-smooth muscle actin (α-SMA) (1:250 dilution; A2547, Sigma) or lysyl oxidase (LOX) (1:50 dilution; sc-373995 Santa Cruz). Briefly, cells were cultured on coverslips, fixed in 1% paraformaldehyde for 20 minutes and permeabilized with 0.2% Triton-X100. The blocking step was performed using 2.5% BSA in 1X PBS for 30 minutes. Then the cells were incubated with primary antibodies for 2 hours at room temperature followed by washes in PBS and incubation with the secondary antibody (Alexa 488, 1:500 dilution; Thermofisher Scientific) for 1 hour. Imaging was performed using a Zeiss Axioplan microscope with Axiocam HrC camera.

For the images in Figure 1A, the co-cultures were fixed and labelled with F-actin.

**Three-dimensional collagen matrices (3-DCM)**

The ability of different Fb phenotypes to contract the extracellular matrix (ECM) was measured using unrestrained 3-DCM. An equal number of cells was mixed in rat tail collagen type I (1.5 mg/ml; Corning) and Dulbecco’s Modified Eagle Medium (DMEM) (Sigma). The collagen cell mixture was transferred to a 1% BSA coated 24-well plate and incubated at 37°C. After one hour, wells were filled with fresh DMEM without fetal bovine serum (FBS) and imaging was performed after 48 hours. The total contraction of the gel was evaluated by measuring the diameter of the gel using ImageJ software.

**Sircol collagen assay and Sirius red staining for collagen quantification**

Collagen secreted by different Fb phenotypes in culture was quantified using the sircol collagen assay (Biocolor). Briefly, after 6 days in culture, conditioned medium and cell pellets were harvested from different Fb cultures. Intracellular and extracellular collagen production from respectively the cell pellet and the conditioned medium were measured. Fb cultures on coverslips were stained for collagen using the PicroSirius red staining kit (PolySciences). Imaging was done using a Zeiss Axioplan microscope with the Axiocam HrC camera. All experiments were performed according to the manufacturer’s instructions.

**Western blot analysis**

Whole cell protein lysate was prepared using RIPA buffer, as explained previously^1^. The equal amount of protein was separated using NuPAGE™ 4-12% bis-tris protein gel (ThermoFisher Scientific) and transferred to a PVDF membrane. The blot was incubated with a rabbit polyclonal anti-Cx43 antibody (C6219, Sigma-Aldrich) overnight. After three times wash, the blot was incubated with anti-rabbit IgG HRP-linked secondary antibody (7074, Cell Signalling technology) and developed with SuperSignal™ west femto maximum sensitivity substrate (ThermoFisher Scientific). The upper part of membrane was cut and re-probed with calnexin antibody (C4731, Sigma-Aldrich) and used as loading control. The blot was imaged and analysed using Image lab TM analyser software (Bio-Rad laboratories N.V.). The original blot image is added in supplemental figure S5.


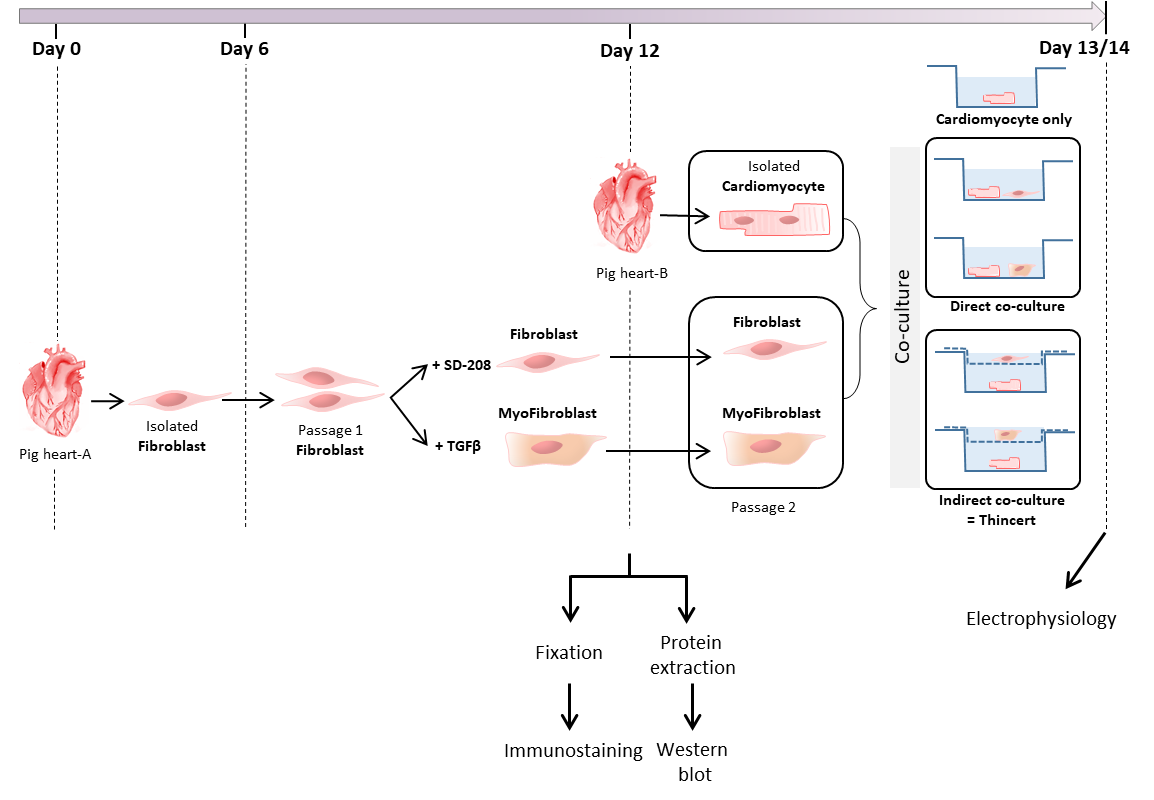


**Supplemental Figure S1: Experimental design.** Left ventricular Fbs were isolated and cultured for 6 days. At day-6, when they are 80-90% confluent, Fbs are trypsinized and split. From day-7 until day-12, cultured Fbs are treated either with SD-208 or with recombinant TGF-β1. At day-12, Fbs were fixed and used for immunostaining analysis. Also on day-12, CMs were isolated from another pig and pre-plated in a culture dish. After 3 hours, the supernatant containing CMs were collected and re-plated onto laminin coated coverslips in a 6-well plate. To this 6-well plate containing CMs, different Fb phenotypes were added either directly or to a thincert, which was placed within the 6-well plate, as shown in the figure. The CM-Fb co-cultures were used for structural and electrophysiological studies.

Supplemental data


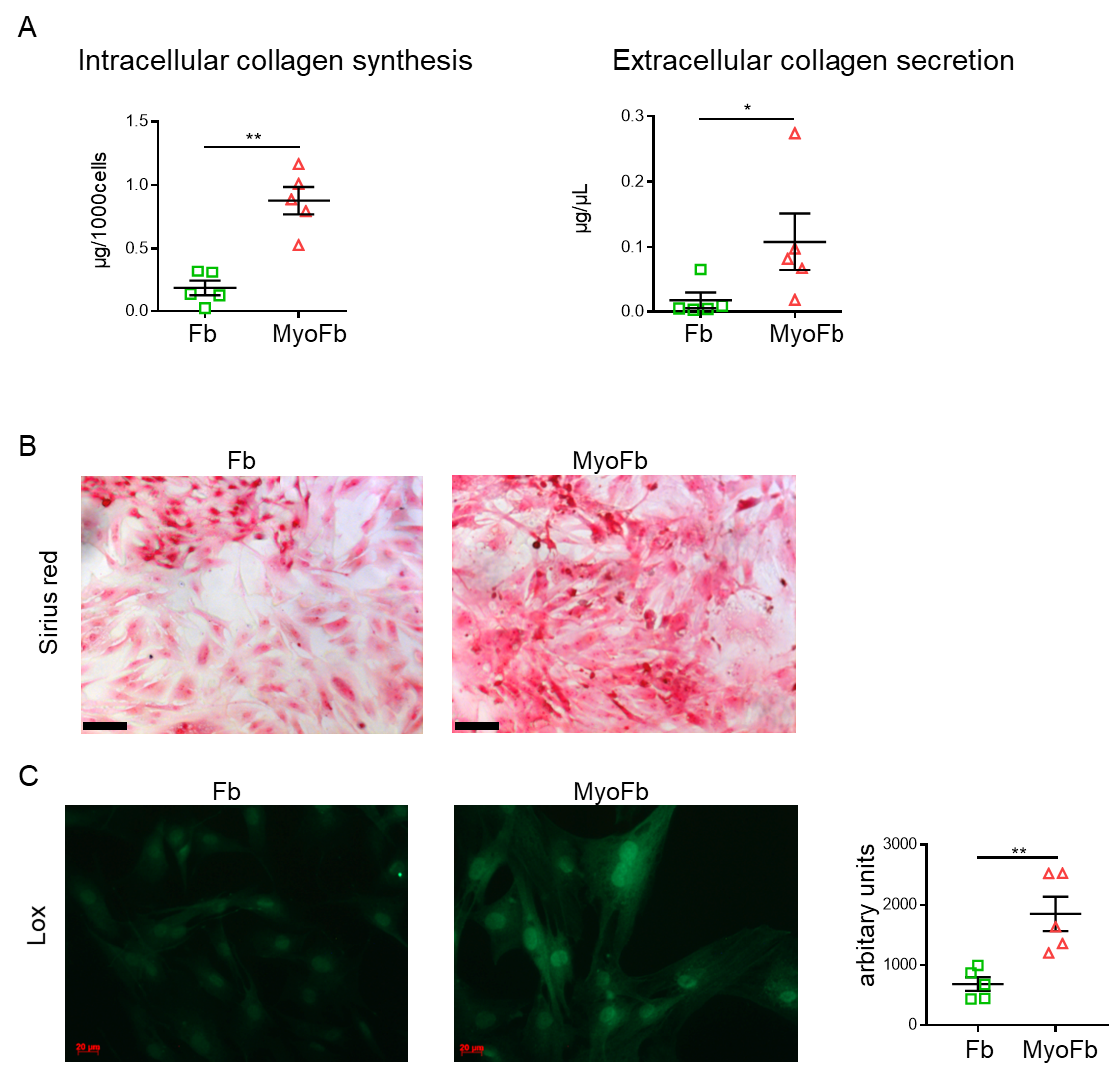


**Supplemental Figure S2: Increased collagen synthesis and cross-linking by MyoFbs.** (A) Intracellular collagen and extracellular collagen measured in Fb cell lysate and conditioned medium respectively. (B) Sirius red staining of Fbs. (C) Immunostaining and quantification of LOX in Fbs. Scale bar represent 20 μm. (Unpaired Student t-test). (*p < 0.05: **p < 0.01).

coverslip

B

A


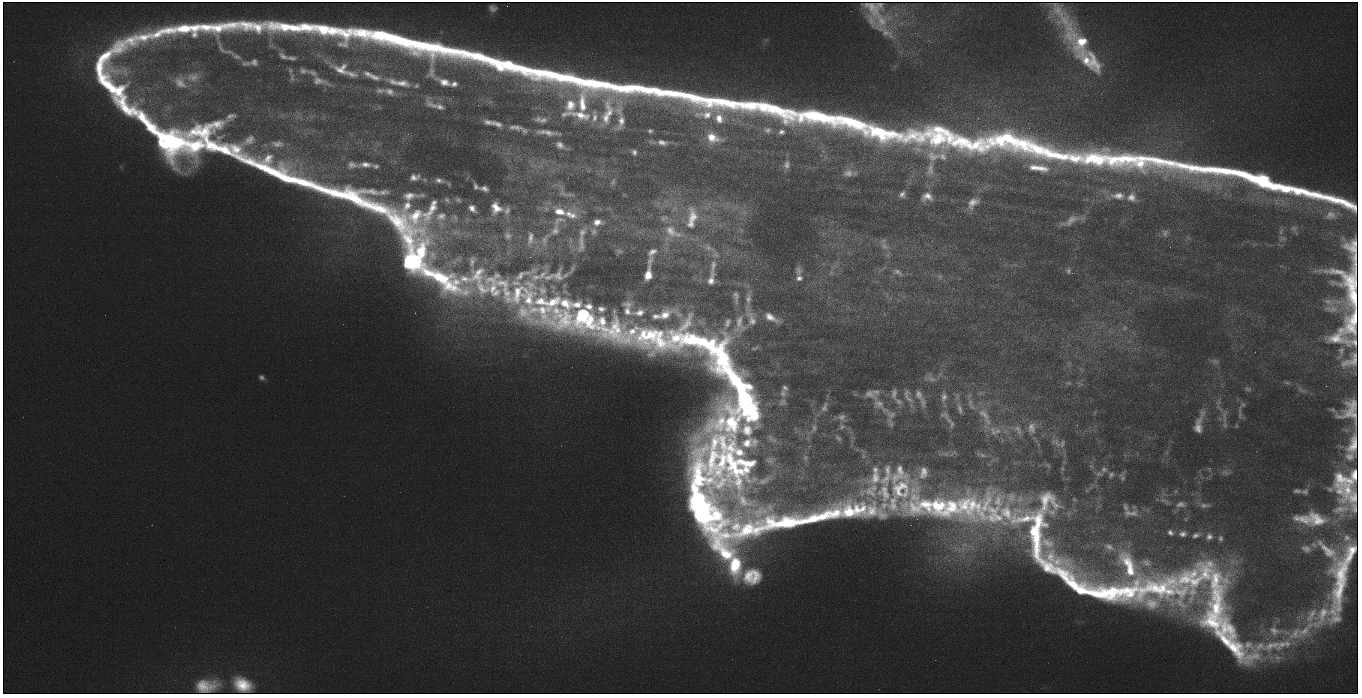

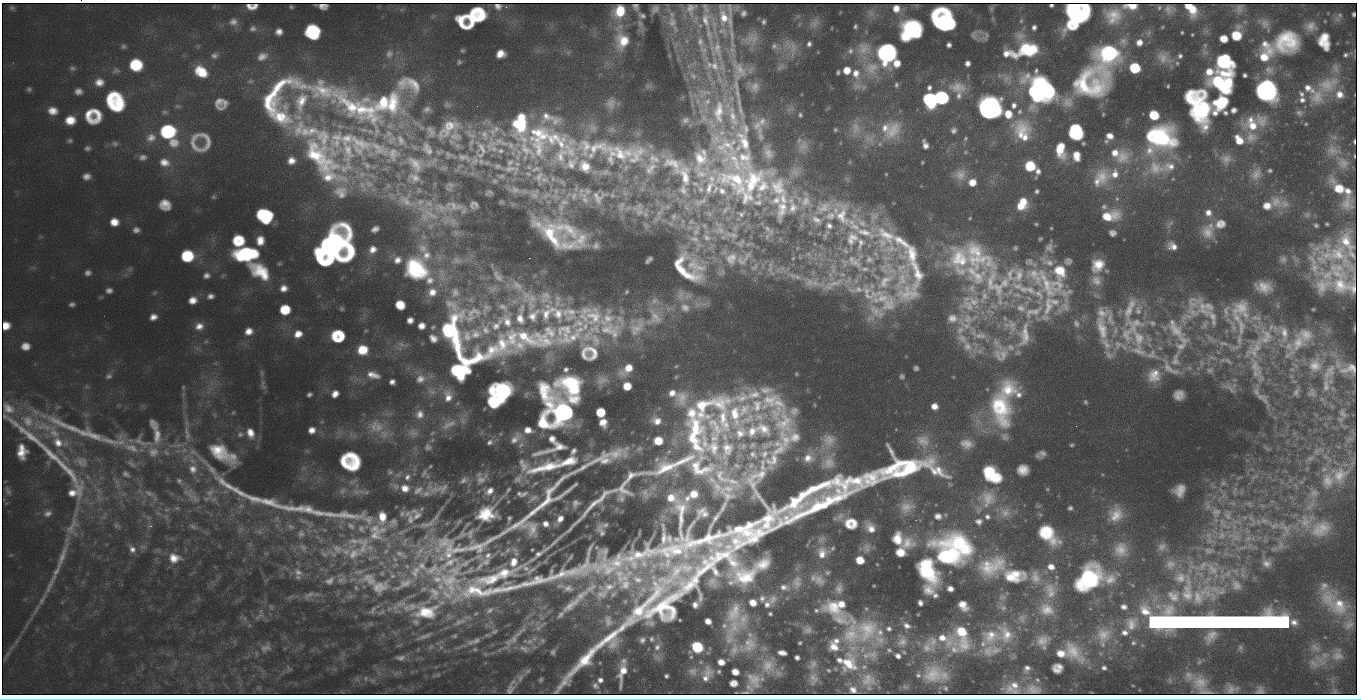


↑+10 µm

**Supplemental figure S3. Measuring the transverse and axial tubular network (TATS).** (A) Quantification of TATS of CMs at Day-0, Day-1 and Day-2. (1-way ANOVA with Bonferroni post hoc test). (B) Representative confocal images of membrane stained myocytes (WGA) in co-culture with activated MyoFbs. On the coverslip plane (top image), protrusions from the MyoFb allow direct contact between the two cell types. On an upper plane (bottom image), only myocytes (and their TATS) are visible. Scale bar 20 μm.


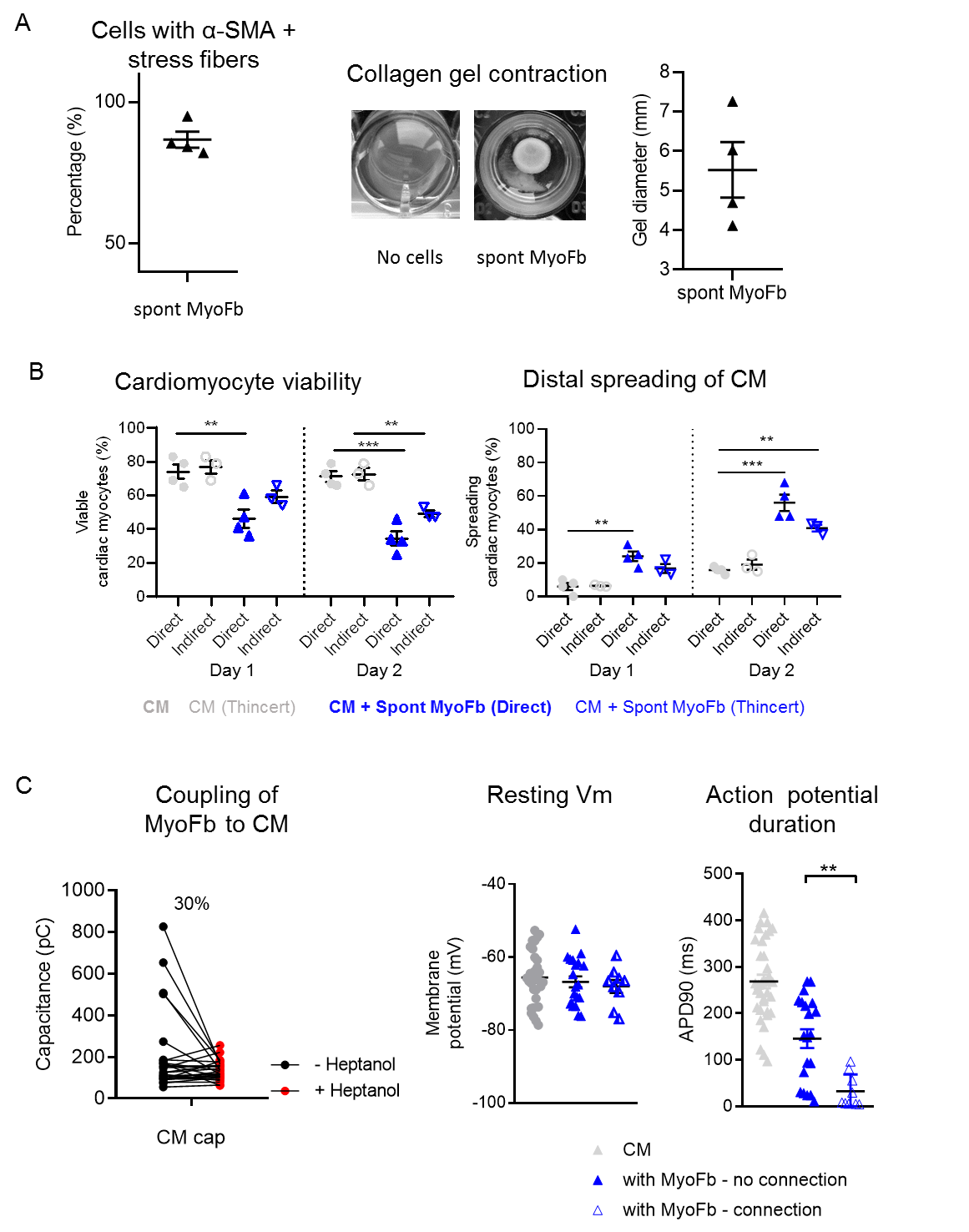
**Supplemental Figure S4.** **Spontaneously differentiated MyoFbs have similar properties and effects as compared to TGF-B1 treated cells.** Cells were cultured from day-7 until day-12 without any treatment and subsequently put into co-culture with CMs, as in Figure S1; these cultures were done in parallel with the main data. Spontaneously differentiated MyoFbs are very similar to the TGF-β1 treated MyoFbs, with presence of contractile stress fibers (A), reducing myocyte viability and enhancing spreading in direct co-cultures (B). They also readily make electrical couplings that shorten the APD (C) (1-way ANOVA with Bonferroni post-hoc test, ** p = 0.0036). For comparison, CM data are included (grey symbols).

**
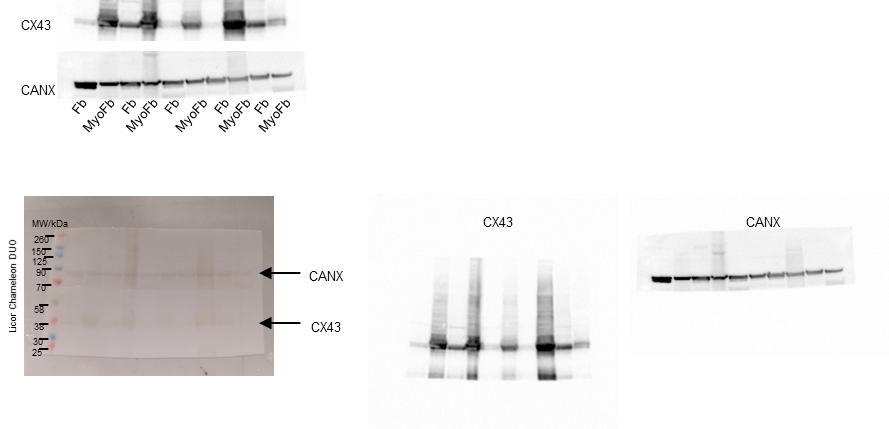
**

**Supplemental Figure S5.** Uncropped blot image.

**References**

1. Wang, N. *et al.* Connexin mimetic peptides inhibit Cx43 hemichannel opening triggered by voltage and intracellular Ca2+ elevation. *Basic Res. Cardiol.* **107,** 304 (2012).

2. Adam, O. *et al.* Increased lysyl oxidase expression and collagen cross-linking during atrial fibrillation. *J. Mol. Cell. Cardiol.* **50,** 678–85 (2011).
